# Diseases of the Antrum

**Published:** 1890-01

**Authors:** William Carr

**Affiliations:** New York City


THE DENTAL REGISTER.

Vol. XLIV.] JANUARY, 1890. [No. 1.

### Communications.

### Diseases of the Antrum.

BY WILLIAM CARR, M.D., D.D.S., OF NEW YORK CITY.

Read in the Section of Oral and Dental Surgery, at the Fortieth Annual Meeting of the American Medical Association, June, 1889.

In the preparation of this paper, it was not deemed necessary to describe the anatomy of the maxillary sinus or to mention all its pathological conditions, as the principal end in view was to present in a practical manner a few observations on that form of antral disease that is of especial interest to us as oral surgeons, namely, suppuration. Different authors vary in opinion regarding the causes leading to suppuration. The throat specialist and the dentist view this disease from the standpoint with which each is most familiar, and it is difficult to form an accurate estimate of the specific causes that produce the greatest number of cases. Zuckerkandl, in his treatise on "The Normal and Pathological Anatomy of the Air Passages," claims that a majority of the cases of suppuration of the maxillary sinus, directly result from "the extension of the inflammatory process of the nasal mucous membrane, produced by continuity of tissue." That is to say, that rhinitis, either chronic or acute, may cause an inflammatory condition of the sinus and consequent suppuration. Zuckerkandl further says: "My experience with the inflammatory diseases of the lining of the maxillary sinus is, that they mostly follow pathological processes of the nasal mucous membrane, and accordingly the soft parts of the nasal and maxillary cavities are generally diseased together." This view is coincided with by a number of authors. From the advance sheets of Dr. B[illegible]sworth's new work on diseases of the nose and throat, I notice that he holds different views. He maintains that few, if any, cases can be traced directly to this cause, claiming that inflammation of the mucous membrane shows but slight tendency to extend from one anatomical region to another. This is doubtless true—we have all observed that an inflammatory condition of the mucous membrane of the oral cavity, although severe, seldom extends to other anatomical regions. As an argument to sustain his theory, Dr. Bosworth states that "but few persons suffer from antral disease compared with the great numbers who are affected with chronic rhinitis." At the same time, he admits that, in many cases, hypertrophic rhinitis produces antral disease—not by extension, but, because this hypertrophic process, in some manner, causes occlusion of the ostium maxillare.

Watson, of London, on "Diseases of the Nose," expresses the opinion that nasal polypi are the most frequent causes of suppuration of the maxillary sinus, from the fact that, in nearly every case, they originate near the ostium maxillare, and, as they develop, they produce an occlusion of the orifice.

Boerhave's researches show that "in its normal condition, the antrum contains a bland, inodorous, gelatinous, colorless fluid;" that this secretion keeps the walls moist, but does not accumulate in the sinus, it being partly absorbed, and, possibly, partly evaporated by the passing air current.

But, should causes operate to close the orifice of the sinus, the external air could not penetrate into it, nor could the air already in the cavity escape from it. In this case, the vascular system would act as a medium for the gradual absorption of the confined air, which would, necessarily, be replaced in the sinus by the secretion. But, as this secretion could not be absorbed as rapidly as exuded, the air yet remaining in the cavity of the sinus would decompose the accumulated mucus, thereby causing irritation and disease of the mucous membrane.

Thus, by closure of the ostium maxillare, it is possible for the antrum to become diseased, and with its increased secretion, to produce ectasis of the sinus.

From this we can readily understand how nasal polypi and chronic rhinitis may, in many cases, produce antral disease. While I admit that antral diseases are produced by these causes, yet I am convinced from the number of cases that have been under my immediate observation, that fully 80 per cent. of the cases of suppuration of the antrum are caused either directly or indirectly by diseased teeth. When we consider the anatomical relation of the teeth to the antrum, that they are separated only by a thin layer of bone, and, frequently, the roots of the teeth form protuberances in the floor of the antrum, or actually penetrate into it; when we consider the vascularity of the alveolar process, and the frequent pathological changes of the teeth and their alveoli—these changes being pericementitis, alveolar abscess, alveolar periostitis and necrosis—we can easily comprehend why diseased teeth cause such a large percentage of antral disease.

The symptoms of antral suppuration vary with the exciting cause. When the ostium maxillare is closed either by hypertrophy, nasal polypi or inspissated mucus, the first symptom may be only slight febrile disturbance; but the disease is often ushered in with intermittent fever and rigors accompanied by violent pains, a sense of weight and pressure in the sinus, with distension of its walls, soreness of the teeth and œdema of the cheek. If the suppuration is due to defective teeth, the above symptoms are usually absent, excepting the copious discharge from the nostrils. The natural orifice into the meatus is usually found unobstructed, and the mucous membrane of the facial surface greatly congested as in severe cases of pericementitis. After diagnosis has been established, the treatment of antral suppuration is simple in its character. If caused by obstruction of the ostium maxillare, the obstruction should immediately be removed and an effort made to effect a cure through the nares. Should this be unsuccessful, the question arises: Where shall an opening be made for evacuating the sinus of the accumulated secretions, and for subsequent treatment? Various operations have been suggested for this purpose. Owing to a natural hesitancy to remove sound teeth, it has been suggested that an opening be made in the meatus between the inferior turbinated bone. I

believe this operation is unjustifiable. While the opening may be made without difficulty, it would be repugnant to the patient, the operation seeming to the patient to be more formidable than it really is. Neither would it afford the same facilities for thorough cleansing as would an opening through the alveolus. I should never hesitate to make an opening through the alveolus even at the sacrifice of a sound tooth, and, in such a case, should be governed in my choice of extraction by the character of the tooth, always selecting the weakest. By preference, I should extract the second molar, as this makes an opening nearer the centre of the floor of the antrum; but if either the first or the third molar, or the second bicuspid is defective, I should remove it. Should it seem best to extract the second bicuspid, I should drill upward and slightly backward, as, at this point, the floor of the antrum is thicker than at the other points mentioned. In case the teeth on the affected side are all missing, I should select the point corresponding to the second molar. If the trouble arises from decayed teeth and necrosis is present, all necrosed bone should be removed. Before commencing either to remove necrosis or to make an opening into the antrum, a 10 per cent. solution of cocaine should be applied to the gums three or four times in order to produce local anæsthesia of the mucous membrane. The operation is then performed with but slight inconvenience or pain to the patient. The opening being established the antrum should be thoroughly cleansed with tepid distilled water, to which a little salt has been added, until all traces of pus have disappeared. If the discharge is offensive, the cavity should be syringed with permanganate of potash, and dressed with a stimulating solution composed of carbolic acid 1 drachm, glycerine 1 ounce, and distilled water 7 ounces. Or, if preferred, Dobell's solution or listerine may be applied. The cavity should be cleansed twice daily with salt water, followed by either of these stimulating solutions until the discharge diminishes, when this treatment once daily will be sufficient. Should the orifice in the meatus be closed by inspissated mucus, the patient should be instructed to use either of the above stimulants several times daily, by means of a nasal spray, until the obstruction is emoved.

Some practitioners insert a silver drainage tube through the orifice, which is kept in place by ligatures. I see no advantage to be derived from this practice, as thorough cleansing twice daily is all that is required. Besides, there is always the possibility of food being pressed through the tube into the sinus, or of becoming clogged at the entrance, so that the end sought is not attained. I prefer the method suggested by Dr. Abbott, of closing the opening by means of a broom straw, serrated and wrapped with carbolized cotton, then pressed firmly into place, and retained with ligatures to the teeth. If there are no contiguous teeth, a plate may be inserted with a projection one-half the size of the orifice, and this projection wrapped with carbolized cotton to fill the remaining space. This is sufficient to exclude all foreign substances. After the discharge ceases, the cotton should be diminished in quantity daily, thus permitting the orifice to close gradually. This is the only treatment that I have used, and it has been uniformly successful. The following is a brief history of a few typical cases that I have treated :

Case 1.—Mr. M., æt. 33, who stated that, until within two years, he had been in perfect health. He then noticed an offensive discharge from the right nostril which usually disturbed him greatly upon retiring, causing violent coughing when lying upon the left side. Also, upon arising in the morning he experienced nausea, which continued until the nasal cavity had been entirely cleared. He supposed that he was suffering from catarrh, for which he sought and received treatment, at intervals, for twenty-two months, when the following additional symptoms were manifested : At intervals of three or four days he experienced attacks of vertigo, followed by severe otalgia, and great tenderness of the teeth. For these symptoms he was treated by his family physician for three months, who finally advised him to consult me regarding what he supposed to be an alveolar abscess. The right side of his face was then greatly swollen. Upon examination the full number of teeth were found, but the second bicuspid had been filled and was pulpless. My diagnosis was not that of simple alveolar abscess, but of suppuration of the antrum. The extraction of the second bicuspid was followed by a slight flow of pus. A further examination showed the alveolar process greatly necrosed, but there was no visible opening into the antrum. After an application of cocaine all necrosis was removed and an opening made into the antrum, when a great quantity of offensive matter escaped. The lining membrane of the antrum had thickened to at least ten times its normal thickness; this pathological condition I have found in all chronic cases upon which I have operated. Upon proceeding to syringe the sinus, I failed to establish an outlet through the opening into the nares. The sinus was first cleansed with salt water, then with permanganate of potash, after which the orifice was closed in the manner already described. The patient was then directed to use Dobell's solution by means of a nasal spray in order to remove any secretion from the nares. The following day the pledget of straw and cotton was removed, when the discharge seemed greater than on the previous day. The opening through the meatus had by this time been establisned, and the cavity was thoroughly syringed with hot water until all traces of pus had disappeared. Then it was cleansed with a stimulating solution and the pledget removed. This treatment was continued daily for three months, when the case was cured and the patient dismissed. I have seen him since, at intervals during the past three years, and there are no signs of recurrence of the disease.

CASE 2.—Miss J., æt. 30, had considerable discharge from the right nostril, and had been treated for catarrh. The right side of her face was greatly swollen. Upon the affected side were found the second and third molars, the first bicuspid, and the canine in a healthy condition. The first molar and second bicuspid had been extracted for alveolar abscess three years previously. The swelling of the face had appeared twice before, for which she had been treated by her family physician. After lancing, a free discharge of pus followed, and an opening was found into the antrum. After the usual treatment for five weeks the discharge ceased, all parts resumed their normal condition, and the opening in the antrum was allowed to close. In this case the suppuration was caused by one or both of the teeth previously extracted for alveolar abscess.

CASE 3.—Mrs. P., æt. 45 years, who had been suffering for several years with intermittent fever, and had also been for some time troubled with a discharge of an offensive nature from the left nares which had been diagnosed as catarrhal, called to consult me about a first molar. Examination showed that all the teeth, from the canine including the third molar, had been removed excepting the first molar. This was badly decayed, loose, and upon pressure, pus oozed from the socket. The tooth was extracted, disclosing a cavity into the antrum corresponding to the roots of the teeth. The extraction was followed by a copious, purulent, offensive discharge. There was considerable necrosis present. After treatment for over two months without any perceptible improvement, the patient left the city for the summer and was under treatment while absent. At the expiration of four months, she returned, yet without improvement. Upon a thorough examination, a septum of bone, about one-quarter of an inch in height was found rising from the floor of the antrun, dividing the cavity into two parts. By means of a chisel, this septum was removed and found to be composed of cancellous tissue. After this operation, the case readily yielded to treatment, and a recovery resulted.

Case 4.—Mr. J., æt. 23 years. In this case there was no nasal discharge, the orifice from the sinus being closed by inspissated mucus. He complained of great weight and pressure on the affected side, severe otalgia and deafness. The second bicuspid was missing, and the first molar having a large amalgam filling, was pulpless. This tooth was extracted leaving a large opening into the antrum. There was also considerable necrosis, which was removed, and the usual stimulating treatment pursued for about four weeks, when the discharge ceased, and all other abnormal symptoms disappeared excepting the deafness.

Miss S., æt. 19 years, consulted me with regard to a large swelling of the right side of her face. Before examination, I supposed that she was suffering from alveolar abscess; but to my astonishment upon examination, all her teeth were found in a healthy condition and the arch appeared unbroken. This clearly did not indicate alveolar abscess, but rather a diseased antrum. As the teeth all seemed to be present, I inquired whether she had previously received a blow upon that side of her face, or whether she had not recently contracted a severe cold. Receiving a negative reply to these questions, I made a second examination and found that the first bicuspid was missing. She positively assured me that none of her teeth had been extracted. As the swelling pointed to the palatine as well as the facial surface, I lanced both surfaces, and was enabled to pass a probe from the facial surface into and through the floor of the antrum. She was dismissed until the next day, when I made a careful exploration of the sinus by means of an excavator, and found in the floor of the antrum the missing tooth imbedded in the inner coat of the mucous membrane, its apex toward the alæ. After the swelling subsided, the tooth was dislodged by means of a hoe excavator, and removed by means of a long, narrow-beaked forcep. After four weeks' treatment the case was cured.

During the past eight months, in addition to the cases in my own practice, I have operated upon fourteen cases of diseased antrum for a throat and nose specialist. Of these cases none resulted from chronic rhinitis, none from hypertrophic rhinitis, one from dentigerous cyst, two from polypi and eleven from diseased teeth. This seems to me clearly to refute the assertions of the authorities previously mentioned—that suppuration of the antrum is generally due to causes other than diseased teeth.

